# Supplementary material for: Polyandry contributes to Gonipterus platensis (Coleoptera: Curculionidae) rearing
Source: PeerJ. 2024 Aug 22;12:e17929. doi: 10.7717/peerj.17929 (PMC11344996; doi:10.7717/peerj.17929)
Supplement: Supplemental Information 1 — The abstract indicates that in a experiment for Gonipterus platensis rearing the Polyandry with choice treatment resulted in the longest period of oviposition, highest fecundity and highest number of eggs per egg capsules when compared to monoandrous females. The polyandry with no choice treatment didn’t differ from monoandrous or polyandry with choice treatments in almost all parameters, presenting intermediate values among those treatments. The pre-oviposition period, fertility and effect of the treatments on progeny were not significantly affected. [file peerj-12-17929-s001.pdf]

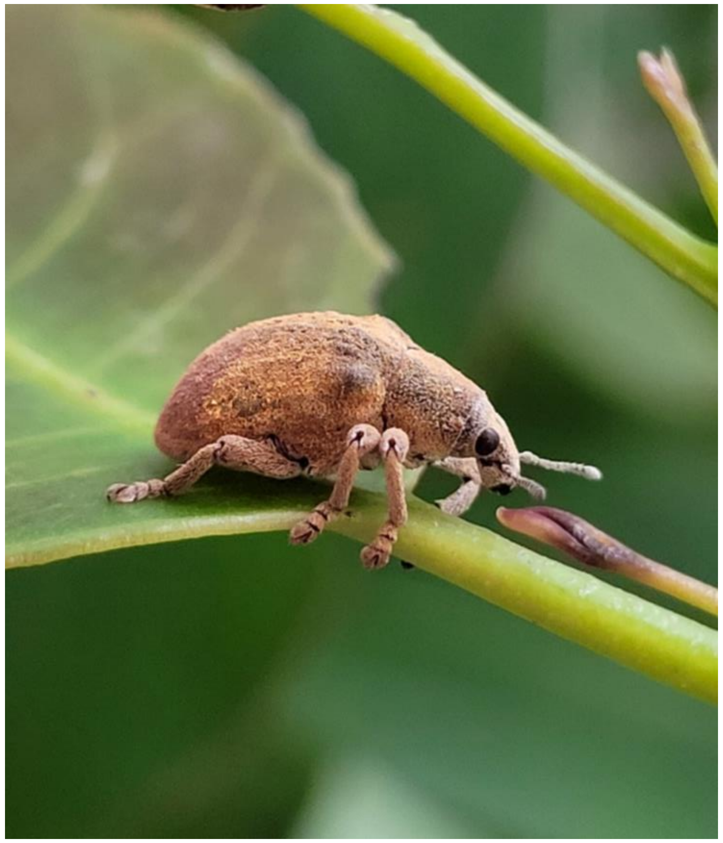

	Monoandry	Polyandry no choice	Polyandry with choice
Pre-Oviposition period	–	–	–
Oviposition period	✗	◀▶	✓
Fecundity	✗	◀▶	✓
Egg capsule size	✗	✓	✓
Fertility	–	–	–
Efect on progeny	–	–	–

✗ Negative influence   – No influence   ◀▶ Not stastitically different   ✓ Positive influence
