# Supplementary material for: Polyandry contributes to Gonipterus platensis (Coleoptera: Curculionidae) rearing
Source: PeerJ. 2024 Aug 22;12:e17929. doi: 10.7717/peerj.17929 (PMC11344996; doi:10.7717/peerj.17929)
Supplement: Supplemental Information 2 [file peerj-12-17929-s002.docx]

SM1 - Number of matings per female, pre-oviposition, and oviposition period (days) (Mean ± SE) of Gonipterus platensis (Coleoptera: Curculionidae) for monoandry, no choice polyandry and polyandry with choice treatments. T=25±1ºC; RH 60% and photoperiod 12:12h

| Treatment | N* | Number mating | Pre-oviposition | Oviposition |
| --- | --- | --- | --- | --- |
| Monoandry | 9 | 2,44 ± 0,41 ab | 45,44 ± 8,66 a | 76,56 ± 22,34 b |
| Polyandry no choice | 7 | 1,29 ± 0,18 b | 40,86 ± 13,02 a | 109,06 ± 23,06 ab |
| Polyandry with choice | 9 | 4,11 ± 0,96 a | 34,67 ± 9,81 a | 151,33 ± 16,99 a |

Means followed by the same letter on the column did not differ by the Tukey-Kramer test (p<0,05).

N* = number of replications

SM 2 - Number of eggs, percentage of infertile eggs and number of eggs per egg capsule (Mean ± SE) of Gonipterus platensis (Coleoptera: Curculionidae) on monoandry, no choice polyandry and polyandry with choice treatments. T=25±1ºC; RH 60% and photoperiod 12:12h

| Treatment | Number of eggs | Infertile eggs (%) | Eggs/Egg capsule |
| --- | --- | --- | --- |
| Monoandry | 145,56 ± 53,94 b | 6,30 ± 1,78 a | 3,67 ± 0,48 b |
| Polyandry no choice | 240,29 ± 61,78 ab | 7,05 ± 1,32 a | 4,95 ± 0,26 a |
| Polyandry with choice | 361,00 ± 76,95 a | 8,31 ± 2,34 a | 5,28 ± 0,36 a |

Means followed by the same letter on the column did not differ by the Tukey-Kramer test (p<0,05).
